# Identification of recurrent regions of copy-number variants across multiple individuals

**DOI:** 10.1186/1471-2105-11-147

**Published:** 2010-03-22

**Authors:** Teo Shu Mei, Agus Salim, Stefano Calza, Ku Chee Seng, Chia Kee Seng, Yudi Pawitan

**Affiliations:** 1 Department of Epidemiology and Public Health, National University of Singapore, 16 Medical Drive, 117597, Singapore; 2 Centre for Molecular Epidemiology, National University of Singapore, 30 Biopolis Street, 138671, Singapore; 3 Department of Biomedical Sciences and Biotechnology, University of Brescia, Viale Europa, 11 25123 Brescia, Italy; 4 Department of Medical Epidemiology and Biostatistics, Karolinska Institutet, Nobels väg 12A, Stockholm 17177, Sweden; 5 NUS Graduate School for Integrative Sciences and Engineering, National University of Singapore, 28 Medical Drive, 117456, Singapore

## Abstract

**Background:**

Algorithms and software for CNV detection have been developed, but they detect the CNV regions sample-by-sample with individual-specific breakpoints, while common CNV regions are likely to occur at the same genomic locations across different individuals in a homogenous population. Current algorithms to detect common CNV regions do not account for the varying reliability of the individual CNVs, typically reported as confidence scores by SNP-based CNV detection algorithms. General methodologies for identifying these recurrent regions, especially those directed at SNP arrays, are still needed.

**Results:**

In this paper, we describe two new approaches for identifying common CNV regions based on (i) the frequency of occurrence of reliable CNVs, where reliability is determined by high confidence scores, and (ii) a weighted frequency of occurrence of CNVs, where the weights are determined by the confidence scores. In addition, motivated by the fact that we often observe partially overlapping CNV regions as a mixture of two or more distinct subregions, regions identified using the two approaches can be fine-tuned to smaller sub-regions using a clustering algorithm. We compared the performance of the methods with sequencing-based results in terms of discordance rates, rates of departure from Hardy-Weinberg equilibrium (HWE) and average frequency and size of the identified regions. The discordance rates as well as the rates of departure from HWE decrease when we select CNVs with higher confidence scores. We also performed comparisons with two previously published methods, STAC and GISTIC, and showed that the methods we consider are better at identifying low-frequency but high-confidence CNV regions.

**Conclusions:**

The proposed methods for identifying common CNV regions in multiple individuals perform well compared to existing methods. The identified common regions can be used for downstream analyses such as group comparisons in association studies.

## Background

Copy-number variants (CNVs) are genomic regions that contain an abnormal number of copies. In humans, we normally expect two copies of each autosomal region, but in CNV regions we may observe copy gains or losses. Current common technology used for CNV detection are high-density single nucleotide polymorphism (SNP) arrays or array comparative genomic hybridization (aCGH) arrays. Detection of CNVs from aCGH arrays is mostly based on locating change-points in intensity-ratio patterns that would partition each chromosome into several discrete segments [1-5]. On the other hand, the hidden Markov model (HMM) is particularly popular for detection of CNVs from SNP arrays, where the hidden states provide a natural way of combining information from the total signal intensity and the allele frequency values (see for example, [6,7]). These approaches detect CNVs sample-by-sample, and because of the high noise level in the intensity values, especially for SNP array data, the boundaries of the detected CNVs tend to vary among individuals. However, in a homogenous population, common CNV regions are likely to occur at the same genomic locations across different individuals. Our focus in this paper is to identify common CNV regions in multiple individuals from a given population.

Common CNV detection algorithms for SNP arrays report the log Bayes factor as a confidence score for each identified region; this provides a measure of the reliability of a detected CNV within an individual. Previous methods developed to identify recurrent CNV regions (see [8] for a review) were primarily developed for aCGH data and hence did not incorporate confidence scores. For example, a previously published method, STAC [9], uses two statistics to identify recurrent CNV regions. These statistics are based on the frequency of occurrence of the regions and the alignment of the regions. However, since the method does not incorporate confidence scores, every individual region contributes equally to the statistic, whereas in fact, inter-sample variability is bound to exist, where some regions are more likely to be true/false positives. Furthermore, STAC requires each chromosome to be split into non-overlapping windows of a user-defined fixed size. The algorithm then searches for evidence of common CNV regions within each window. The weakness of this is that the output from such an approach will only provide evidence of whether each window harbours a common CNV, but will not indicate the breakpoints of the CNV. Although we may decrease the window size to improve the resolution, in practice, doing so will incur an enormous computational burden.

**Figure 1:**
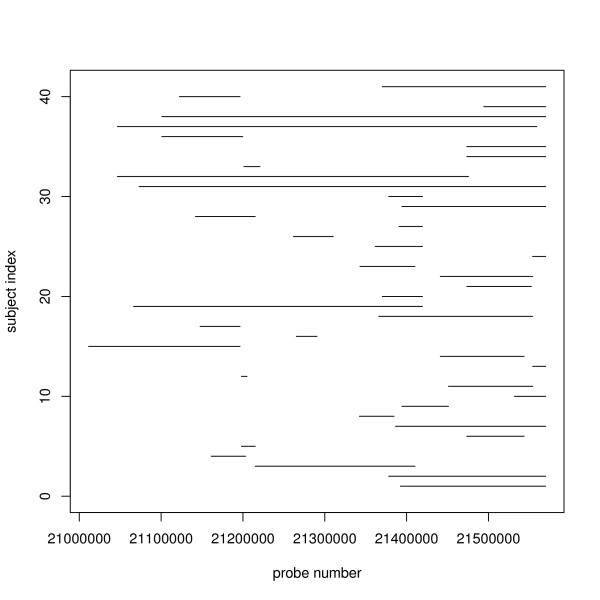
**An example of a common CNV region found based on COVER method with threshold *u *= 2 and *c *= 60**. This figure illustrates a common CNV region in part of chromosome 22, found using the COVER method with threshold *u *= 2 and confidence cutoff at 60th percentile. 41 out of 112 individuals have CNVs that overlap with this common region, indicated by the horizontal lines. We can see that despite being identified as a common region, the individual regions still portray a mixture phenomenon of several distinct subregions.

In this paper, we investigated two different methods to detect common CNV regions. The methods take segmented data as the input. The first method estimates a statistic based on the frequency of occurrence of reliable CNVs, where reliability is determined by a high confidence score. The second method is based on a weighted frequency of occurrence of CNVs, where the weights are determined by the confidence scores. Figure 1 illustrates a common CNV region in chromosome 22, identified using the first method, and shows evidence of several distinct subregions within the identified common region. Hence, in addition to these methods, we also investigated the use of a clustering algorithm to split the common regions into smaller subregions.

To assess the performance of the methods, we ran the algorithms on 112 HapMap samples from the Illumina iControl database, composed of individuals from three populations (Yoruba, Caucasian and Asian). We compared the regions we identified to the regions identified using sequencing [10]. In general, the discordance rates with sequencing-based CNV regions as well as the rates of departure from HWE decreased when we filtered the individuals with a stricter confidence score threshold. To benchmark the proposed methods to currently available methods, we performed comparisons with STAC [9] and GISTIC [11] and found that the proposed methods outperformed both STAC and GISTIC in identifying low-frequency but high-confidence CNV regions.

## Methods

### Data Structure

We assume that the raw intensity data have been processed by a CNV detection algorithm. Denote by *R*_*i *_= {*R*_*i*1_, *R*_*i*2_..., } the collection of CNV regions detected in individual *i*, for *i *= 1,...,*n*. A region is defined by its start and end probe locations, and its CNV type (duplication or deletion). For each region, we assume we have a confidence score statistic that measures the likelihood that the detected region is real. An example of this statistic is the log Bayes Factor (see [6]). For region *j *detected in individual *i*, we denote this statistic as *C*_*ij*_.

### Cumulative Overlap Using Very Reliable Regions (COVER)

Our confidence in a CNV region depends on the within- and between-subject information; our methods shall utilize both information. The within-subject information comes from the strength of the signal within an individual CNV region, and this is measured by the confidence score. The between-subject information comes from the consistency of the CNVs across different individuals. Intuitively, we have less confidence in a CNV that occurs in one individual than one that occurs in many individuals. However, a single occurrence of CNV might still be a true discovery if it is associated with a high confidence score, i.e., it is based on a strong signal.

Since individual CNV regions span different probes, the number of individual regions that overlap each probe varies. However, common CNV regions tend to occur at almost the same genomic locations across multiple individuals. Hence, we expect the common regions to be identified by consecutive probes where a 'significant' number of individuals have an overlapping CNV region. Furthermore, we also expect the confidence score of the individual region to be relatively high.

Let *Z*_*ijk *_be the indicator that region *j *detected in individual *i *overlaps with probe *k*. For each probe *k*, we calculate the Cumulative Overlap using Very Reliable Regions (COVER) statistic *y*_*k*_, defined as

where  is the indicator function for regions detected with a confidence score above a certain threshold *c*. The common CNV regions are then defined by

representing sets of consecutive probes for which *y*_*k *_is consistently greater than or equal to a specified threshold *u*. *l*_*m *_is the genomic position of probe *m *and it is implicitly understood that the cardinal position of the probe reflects its relative position in the chromosome so that when there are *M *probes in a chromosome, *l*_1 _<*l*_2 _<...<*l*_*M*_.

Using COVER, we can identify multiple common CNV regions within a chromosome. Furthermore, different subsets of individuals may contribute to different common regions, hence allowing COVER to identify regions that are common to only a subset of individuals. By only considering individual regions that are detected with high reliability, we also incorporate the uncertainty associated with each individual region in the identification of common regions. If this is not taken into account, then all regions would be treated equally despite the fact that some are more likely to be true than the others. Figure S4 in the [Additional File 1] gives an illustration of how COVER works.

### Cumulative Composite Confidence Scores (COMPOSITE)

In COVER, regions with low confidence are given zero weights and they do not contribute to the COVER statistic. The within-subject confidence is not fully exploited when computing the COVER statistic: regions that are detected with low confidence but nonetheless detected consistently across a large number of subjects might be missed.

This limitation is addressed in the second method. For probe *k *the composite confidence score (COMPOSITE) statistic is defined as,

This formula is in fact similar to COVER statistic, where instead of using the indicator function  as weights, now all detected individual regions contribute to the COMPOSITE statistic, with the amount of their contribution proportional to their confidence scores.

Using COMPOSITE, the common CNV regions are then defined as

representing sets of consecutive probes for which *s*_*k *_is consistently greater than or equal to a specified threshold *v*. Figure S4 in [Additional file 1] gives an illustration of how COMPOSITE works.

### Clustering of Individual CNV Regions within a Common Region (CLUSTER)

Cluster analysis has been used in the analysis of gene expression and aCGH data (see for example, [12-14]). Here, the motivation for CLUSTER stems from the observation that within a common CNV region identified by COVER or COMPOSITE, a complex mixture phenomenon can still be observed (see Figure 1).

**Figure 2:**
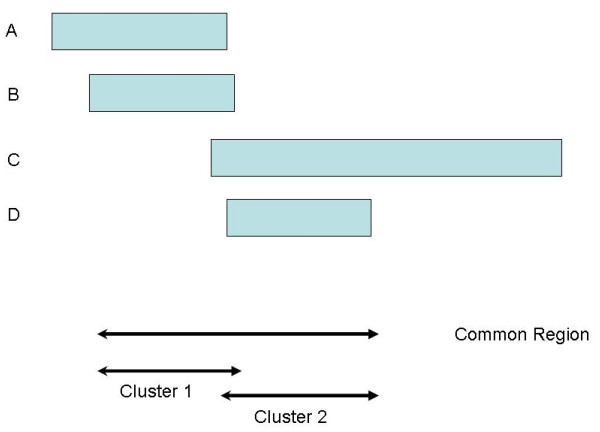
**Hypothetical example of a identified common CNV region with 2 distinct clusters**. Hypothetical situation where an identified common CNV region is common to four individuals. From the figure, it is clear that the common region consists of two partially overlapping regions. The first two individual regions are clustered together to the left of the common region, while the last two individual regions are clustered to the right.

Figure 2 depicts the hypothetical situation where a common region of length *L *bases has been identified by COVER or COMPOSITE. Four individual regions overlap with the common region and from the figure, it is clear that the first two regions are clustered to the left while the last two are clustered to the right. The two groups may form two distinct subregions and these subregions could differ biologically. In reality, the situation is more complex than the hypothetical example here (see for example Figure 1).

To find the subregions inside this common region, we first perform pairwise comparisons of the individual regions that overlap with the common region. For example, the comparison of two regions *A *and *B *can be summarized into 4 values (*a, b, c, d*), where *a *is the number of bases for which both *A *and *B *overlap with the common region, *b *is the number of bases where *A *overlaps with the common region but *B *does not, *c *is the number of bases where *B *overlaps with the common region but *A *does not, and *d *= *L *- *a *- *b *- *c*.

The (dis)similarity index can be computed using a variety of distance metrics appropriate for binary data such as the Manhattan, Canberra or Jaccard distance [15]. The Jaccard distance is particularly attractive for our case; it is defined by *a*/(*a *+ *b *+ *c*) and can be interpreted as the percentage of common overlap of the two regions relative to the union of the overlaps of the two regions with the common region. We then construct a dissimilarity matrix as input to a hierarchical clustering algorithm. The number of clusters will be determined by the amount of within-cluster similarity we require. The boundaries of each subregion will be the minimum and maximum positions of all individual regions that belong in that cluster. If these bounds overshoot the boundaries of the initially identified region, then the boundaries will be reset to the boundaries of the initial region.

## Results and Discussion

### Assessment and Comparison

#### Datasets

We studied the performance of the proposed procedures by varying the corresponding threshold parameters in each approach. 112 HapMap samples, comprising 46 Caucasian (CEU), 29 Beijing Chinese and Tokyo Japanese (CHBJPT) and 37 Yoruban (YRI) individuals were used in the analysis. These samples are part of the Illumina iControl Database. Each sample was genotyped using the Illumina 1M chip, and PennCNV [6] was used to detect the individual CNV regions.

#### Comparison with Sequenced Regions

We compared the common regions we identified to a list of reference CNVs identified in eight HapMap samples using sequencing data [10]. For each of the eight samples, we calculated the discordance rates by recording the proportion of common CNV regions (found using our methods) for that sample that were not concordant with the sample-specific reference CNVs. To be 'concordant' with a reference CNV, a region has to be either contained within the reference CNV or it has to overlap with at least 50% of the reference region. It is important to note however that it is difficult to get a gold standard for common CNV boundaries; even the sequencing-based CNV regions cannot be expected to have 100% sensitivity and specificity in genotype calling and certainly not in boundary calls for common CNVs.

#### Comparison with other Array-based Regions

We compared the regions found using our methods to the regions found by two other groups using array-based methods. We compared with McCarroll *et al*. [16], where the regions were identified using the Affymetrix SNP 6.0 arrays on 270 HapMap samples. To minimize false discoveries, they ran two independent experiments and require a CNV to be observed in both experiments. We also compared our regions to the regions found by Conrad *et al*. [17]. These regions were identified using tiling oligonucleotide microarrays, comprising of 42 million probes, on 41 HapMap samples. A total of 11,700 CNVs were identified, and 8,599 were validated using a set of stringent criteria including (i) additional measurements by Agilent 105K CGH arrays, (ii) overlap with previous studies and (iii) other quality-control filters. For our comparisons, we used only the 8,343 validated CNVs in the autosomal regions.

#### Comparison to other approaches

We compared our approaches to previous common CNV detection methods, STAC: Significance Testing for Aberrant Copy number [9] and GISTIC: Genomic Identification of Significant Targets in Cancer [11].

Briefly, STAC takes segmented data as input and estimates two statistics: 1. A frequency statistic, which estimates the frequency of aberration at each location across all individuals. 2. A footprint statistic, which uses a subset search methodology and counts the number of locations c such that c is contained in a set of intervals (see [9] for more details). It then uses a permutation test to assess the significance of the observed region. STAC requires each chromosome to be split into non-overlapping regions of a user-defined fixed size. The algorithm looks for evidence of common CNV regions within each window, and reports the associated frequency and footprint p-values.

GISTIC first calculates a 'G score' that is associated with both the frequency of occurrence as well as the amplitude of the aberration. Then, it calculates the probability (q-value) of the observed region occurring by chance via a permutation test. One can either input the log intensity ratios, where the GLAD algorithm [18] will be used to segment the data, or input pre-segmented data using other algorithms.

We had also planned to make comparison to another method called MSA [19], but failed because the software, which is part of the GenePattern module, did not work properly. MSA can be viewed as an improvement over STAC, where it extends the notions of frequency and footprint statistics using original intensity ratio data instead of segmented data [8]. We also tried a comparison to RJaCGH [2], which uses a non-homogenous Hidden Markov Model fitted via the Reversible-Jump Markov Chain Monte Carlo method to estimate the probability that a region has copy number alterations; the method also allows the identification of minimal common regions of copy number changes among multiple individuals.

Unfortunately, with our samples, the algorithm did not converge, so we could not proceed with the comparison.

#### Testing Hardy-Weinberg Equilibrium

It has been observed that the majority of common CNV regions are inherited [20]. Hence, for a population of normal (healthy) individuals, we expect, for most of the common regions, the integer copy numbers to be in Hardy-Weinberg equilibrium (HWE). The small number of regions that depart from HWE can be attributed to factors such as recent mutations. For example, McCarroll *et al*. [16] found that about 98% of common diallelic CNV regions do not show significant departure from HWE. In principle, HWE applies to both diallelic CNVs (where only loss or gain of copy numbers are present in addition to normal copies) and multi-allelic CNV regions (where both loss and gain of copies are present).

For diallelic CNVs with only loss and normal-copy numbers (copy-number = 0,1,2), the HWE test can be conducted by treating '0' copies as minor allele homozygous, '1' copy as heterozygous and '2' copies as reference homozygous. Similarly, for CNVs with only gain and normal-copy numbers (copy-number = 2,3,4), we treat '2' copies as reference homozygous, '3' copies as heterozygous and '4' copies as minor-allele homozygous. For multi-allelic CNVs, a model with three or more alleles is needed. However, the HWE test cannot be performed directly on the unphased copy-number because there is an issue with different combinations of alleles producing the same copy-number. For example, in a 3-allele model, a copy-number of 2 can be produced by a combination of '0' and '2' copies or two '1' copy alleles.

When dealing with samples from healthy individuals, we propose to use the outcome of the HWE tests to select 'optimal' parameter thresholds (e.g., *c *in COVER and *v *in COMPOSITE). If we observe a large number of common CNV regions with significant departure from HWE (after accounting for population stratification), it could mean that the parameters we choose are not optimal. When dealing with a mixture of healthy and diseased individuals such as in association studies, it is expected that the CNVs among the diseased individuals will show some degree of departure from HWE as some of the CNVs could be due to recent abberations. We propose performing HWE tests only among the healthy individuals to select the optimal threshold parameters.

## Results

### COVER results

**Figure 3:**
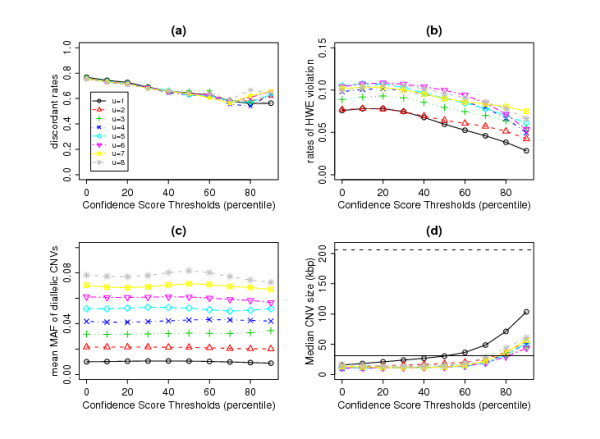
**Results of COVER method**. (a) Discordant Rates, (b) Proportion of diallelic CNVs that failed HWE, (c) mean minor allele frequency (MAF) of diallelic CNVs and (d) Mean CNVs size (kilo-bases) as a function of confidence scores cut-off points and minimum number of individuals.

Figure 3 shows the results for COVER. The discordance rates with Kidd *et al*.'s [10] reference CNVs (see Comparison with Sequencing Results) can be as high as 80% when we include all CNV calls in identifying the common regions. The discordance rates decrease when we exclude CNVs whose confidence scores are below a certain percentile; more severe filtering generally reduces the discordance rates. The lowest discordance rates of about 55% were achieved when we excluded individual regions whose confidence scores were below the 80th percentile. Surprisingly, increasing the required minimum number of individuals inside a region (*u*) does not seem to have an effect on the discordance rates.

However, the required minimum number of individuals (*u*) does affect the rates of HWE violation (calculated as the percentage of diallelic CNVs whose p-value from the HWE test is < 0.01 in at least one of the three ethnic groups). (Some HapMap individuals were related; the HWE test in each ethnic group was carried out on unrelated individuals only.) There is an overall increasing trend for the proportion of common CNV regions that violate HWE when we increase the minimum number of individuals (Figure 3(b)). This is partly due to the fact that with increasing number of individuals, we detect CNV regions with larger minor allele frequencies (see Figure 3(c)), hence the test for HWE will be more powerful. Generally, the rates of departure from HWE are less than 10% and can be lowered by filtering out individuals with lower quality regions. A steeper reduction in the rates of departure from HWE can be observed when only individual regions whose confidence scores are above the 60th percentile are considered (Figure 3(b)).

The sizes of the identified common regions generally increase when we filter lower quality individual regions (Figure 3(d)), reflecting the fact that smaller regions with fewer overlapping probes would tend to have lower confidence scores. By choosing confidence score thresholds (*c*) anywhere up to the 60th percentile, the average size of the common regions are approximately the same or slightly smaller than the average size that Kidd *et al*. [10] obtained using sequencing methods (solid horizontal line in Figure 3(d)). The dashed horizontal line in Figure 3(d) shows that the median size of CNV regions identified using the 500K EA chip [21] is much larger than what we observe using our methods.

For this dataset, setting the confidence score threshold to the 60th percentile seems to be the optimum choice. With this setting, the discordance rates are around 60% and the proportion of diallelic CNVs that violate HWE is kept at around 8%. The choice of *u *is more subjective, as it depends on our definition of 'common' regions. For example, if we require each common region to overlap with at least three individual regions and set *c *to the 60th percentile, we will identify 443 common CNV regions (see [Additional file 2]).

### COMPOSITE results

A total of 89% of the probes does not contain any individual CNV regions and thus their composite scores are zero. So, if we set the threshold *v *at the 89th percentile of the composite scores, we do not filter out any individual regions and this approach is essentially the same as using *u *= 1 and *c *= 0 in COVER.

**Figure 4:**
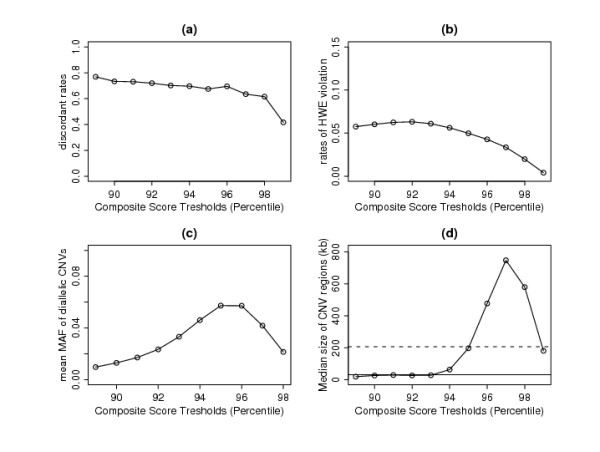
**Results of COMPOSITE method**. (a) Discordant Rates, (b) Proportion of diallelic CNVs that failed HWE, (c) mean minor allele frequency (MAF) of diallelic CNVs and (d) Median size of CNV regions (kb) as a function of composite confidence scores cut-off points. Solid line is median CNV size found by Kidd *et al*.

Figures 4(a) and 4(b) show that, as we increase the threshold, the discordance rates as well as the rates of HWE violation decrease steadily. Unlike the COVER approach, where increasing the confidence score threshold does not result in lower ability to detect rarer CNVs, increasing the composite score threshold does result in fewer rare CNVs being detected (Figure 4(c)). This is because the composite score is a function of both the confidence score and the number of individuals within a common region. By increasing the threshold, we are implicitly requiring more individuals within a common region.

The increasing trend of mean minor allele frequency (MAF) is consistently seen when the threshold is increased to the 96th percentile. Beyond this, the mean MAF decreases because large regions with higher MAF may be split into several subregions with smaller MAF. This observation is consistent with the pattern of median size of CNV regions (Figure 4(d)). Generally, we are losing the smaller regions with low composite scores as we increase the threshold. However, beyond the 96th percentile, the median region size decreases again due to the splitting of the large regions.

The optimal setting is to set the threshold to the 94th percentile, where the proportion of regions that failed HWE is around 5% (Figure 4(c)). Using this setting, we are able to detect 491 CNV regions (see [Additional file 3]) with median CNV size slightly larger then the median size found by Kidd *et al*. [10]. The discordance rates among the eight HapMap samples are approximately 70%, higher than what can be achieved by COVER. Hence, although COMPOSITE can pick up more regions, a higher percentage of these regions is likely to be false discoveries.

### CLUSTER results

The common regions identified using either COVER or COMPOSITE can be further refined into distinct subregions using CLUSTER. Here, we present the results of applying CLUSTER to the common regions identified by COVER. We choose the CLUSTER parameters so that regions will be clustered together if they are at least 60% similar. Complete linkage is used so that the distance between any pair of clusters is defined as the maximum distance between a pair of members drawn one from each cluster. Single or average linkage can also be used. Since single linkage defines the distance between any pair of clusters as the minimum between a pair of members from the clusters, it generally tends to produce clusters that are more similar to each other, and when the same similarity cut-off point is used, it tends to produce fewer clusters than complete linkage. Meanwhile, using average linkage gives more clusters than single linkage, but fewer than complete linkage. In the [Additional file 1], we compare the three linkage measures for a sample region.

**Figure 5:**
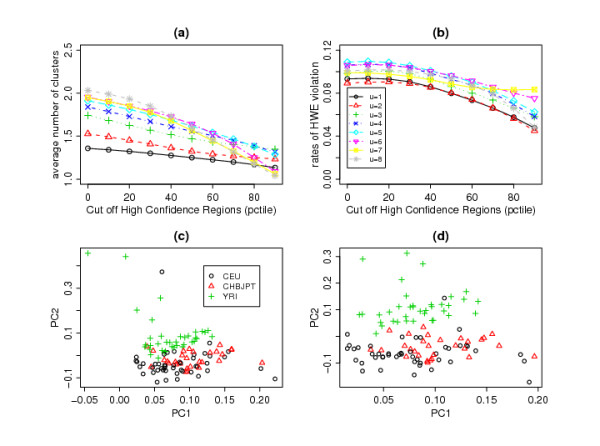
**Results of applying CLUSTER to common regions identified by COVER method**. (a) Average number of clusters, (b) rates of departure from HWE, (c) First and second components of PCA based on subjects' integer copy-number calls at common regions found using COVER (with *u *= 3 and *c *= 60), (d) First and second components of PCA based on subjects' integer copy-number calls at common regions found using complete-linkage CLUSTER (with cluster.limit = 0.6).

Figure 5(a) shows that the number of clusters decreases when we increase the confidence score threshold. But even when we consider CNVs with confidence scores above the median, the clustering effect is still evident with 1.3 to 1.7 clusters found for each common region, depending on which threshold value *u *is used. For the optimum parameters *u *= 3 and *c *= 60, on average, 1.5 clusters are found per common region. The rates of departure from HWE (Figure 5(b)) are approximately the same as in Figure 3(b) and increasing the confidence-score threshold lowers the rates.

Once the common regions are identified, it is straightforward to perform a number of downstream analyses. For example, a principal component analysis (PCA) can done based on subjects' integer copy-number calls at these regions (see Section 'Principal Component Analysis of CNV Profiles' for more details). In the HapMap dataset, CLUSTER clearly improves the separation between the Yoruba and the other two populations based on the subjects' common CNV region profiles(compare Figure 5(c) vs 5(d)). This result suggests that different ethnic groups have more subtle differences in the breakpoints of CNV regions.

#### Comparisons

##### McCarroll et al.'s versus Kidd et al.'s Results

Using the Affymetrix 6.0 arrays, McCarroll *et al*. [16] employed a set of strict criteria based on duplicate experiments to identify the CNV regions. For each of the eight samples sequenced by Kidd *et al*. [10], we calculated the discordance rates with McCarroll *et al*.'s CNVs and they range from 71% for sample NA12878 to 84% for sample NA18517. On average, across the eight samples, 76% of the regions found by McCarroll *et al*. are discordant with the regions found by Kidd *et al*. [10]. In comparison, using COVER, the discordance rates are around 60% (see Section "COVER Results"). Thus, the methods described in this paper, using only data from a non-duplicated experiment, actually perform better in terms of discordance rates against sequencing data.

##### McCarroll et al.'s versus COVER/COMPOSITE Results

**Figure 6:**
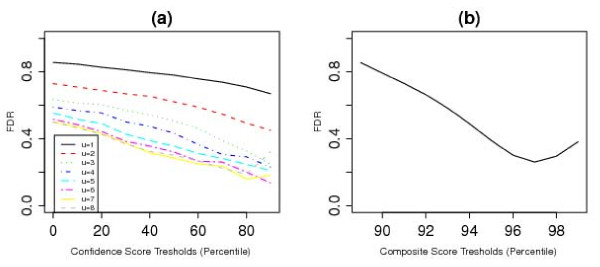
**Comparison to McCarroll's CNVs**. (a) Discordance rates when comparing regions found using COVER and those found by McCarroll *et al*., plotted against confidence score thresholds for different values of *u*. (b) Discordance rates when comparing regions found using COMPOSITE and those found by McCarroll *et al*., plotted against composite score thresholds.

We also compared the regions identified by our approaches to the list of common CNV regions identified by McCarroll *et al*. [16]. Figure 6(a) shows that by using COVER, the discordance rates can be lowered by either increasing the confidence-score threshold, placing a higher limit on the minimum number of individuals (*u*), or both. For the best scenario, the discordance rate is about 15%. Using COMPOSITE, the discordance rates can be reduced by increasing the composite-score threshold, but even for the best scenario, the discordance rate is around 25% (see Figure 6(b)).

##### Comparison to Conrad et al.'s regions

Treating the set of 8,343 validated autosomal CNVs found by Conrad et al. [17] as reference CNVs, we calculate the discordance rates against this reference list. Using the optimal parameters for COVER/COMPOSITE for this dataset, we obtain discordance rates of 42% and 31% for COVER and COMPOSITE respectively. By refining the regions using CLUSTER, the discordance rate for COVER decreases to 34% and that for COMPOSITE remains about the same, at 33%. These are better than McCarroll *et al*.'s [16] regions, which have a discordance rate of 44%.

##### Comparison to GISTIC

As input to GISTIC, we used CNV calls from PennCNV for the same Hapmap samples as described in the Datasets Section. Using the default parameters of GISTIC, with the q-value threshold set at 0.25, we obtained 342 significant common regions with a mean frequency of 0.106 and a median confidence score of 15.7. For comparison with COVER and COMPOSITE, we chose threshold parameters to give the closest number of common regions to that detected by GISTIC. For COVER, this corresponded to the choice of *u *= 3 and *c *= 70th percentile, which yielded 329 regions with a mean frequency of 0.065 and median confidence of 32.3. For COMPOSITE, the threshold was chosen to be the 94.5th percentile, and this yielded 360 regions with a mean frequency of 0.121 and median confidence of 27.6.

For each region identified by COVER, we checked if it was concordant with any region identified by GISTIC. Concordance is defined in the same way as in the Section 'Comparison with Sequencing Results'. The COVER-identified regions can hence be divided into two groups: those that are concordant with at least one GISTIC region and those that are not. For each group, we computed the mean frequency and median confidence score, as well as the discordance rates with Kidd et al.'s regions. We did the same for each region identified by GISTIC, checking if the region was concordant with any region identified by COVER. Similar analysis was done comparing COMPOSITE and GISTIC.

Table 1, for COVER, shows that regions that are concordant with GISTIC regions have higher frequencies but moderate confidence scores, while those that are not concordant with GISTIC regions have lower frequencies but higher confidence scores. The concordant regions have lower discordance rates with sequenced-based results. Similar patterns in frequencies, confidence scores and discordance rates are also seen for the regions found by COMPOSITE. We deduce that GISTIC misses regions that are of low frequencies but high confidence scores. Hence, it seems that COVER/COMPOSITE can identify the low-frequency CNVs better. In addition, of the regions found by GISTIC, those that are concordant with COVER or COMPOSITE have high frequencies and moderate confidence scores while those that are not concordant have low frequencies and low confidence scores. Again, the concordant regions have lower discordance rates with sequenced-based results. From this, we deduce that the regions identified by GISTIC but missed by our methods are those with low frequencies and low confidence scores, and hence more likely to be false positives.

**Table 1:** Comparison with GISTIC.

regions found by	overlap?	no. of regions	mean freq	median conf	discordance**
COVER	✓ GISTIC	139	0.10	30	62%
	✗ GISTIC	190	0.037	37.5	87%

COMPOSITE	✓ GISTIC	162	0.21	20.0	64%
	✗ GISTIC	198	0.048	72.8	75%

GISTIC	✓ COVER	153	0.15	22.3	56%
	✗ COVER	189	0.072	8.8	84%
	✓ COMPOSITE	173	0.15	20.6	61%
	✗ COMPOSITE	169	0.058	8.8	82%

✓ - overlap

✗ - no overlap

** discordance rates with Kidd's sequencing results.

This table shows a summary of the results obtained from comparing COVER/COMPOSITE to GISTIC.

##### Comparison to STAC

For the purpose of analysis using STAC, we split each chromosome into 1450-1500 fixed-size windows with the size of the windows varying from 165 kb for chromosome 1 down to 24 kb for chromosome 22, resulting in a total of 32780 windows across chromosome 1-22. (We tried a smaller window size but the computational burden became too large, where even after 48 hours the algorithm was still running in a 3 GHz windows PC with 4 Gb RAM). We used 0.05 as a cut-off to declare windows with significant frequency or footprint p-values, and obtained 868 significant windows with a mean frequency of 0.155. Each significant fixed-size window will be taken as a significant region.

To compare the regions found by STAC to the regions found using COVER and COMPOSITE, we chose threshold parameters to give a number of common regions closest to that detected by STAC. For COVER, this corresponded to the choice of *u *= 2 and *c *= 60th percentile, and for COMPOSITE, the 93th percentile. We obtained 777 and 805 common regions respectively. We performed similar analysis as in the comparison to GISTIC.

A summary of this comparison is shown in Table 2a. We observe similar results as in the comparison to GISTIC: regions that were identified by STAC but that were missed by COVER/COMPOSITE have low frequencies and low confidence scores, but regions identified by COVER/COMPOSITE that were missed by STAC have low frequencies but high confidence scores, and were thus more likely to be true positives.

**Table 2:** Comparison with STAC.

STAC input: all data regions found by	overlap?	no. of regions	mean(freq)	median(conf)
COVER	✓ STAC	301	0.084	25.6
	✗ STAC	476	0.021	31.2

COMPOSITE	✓ STAC	372	0.14	18.6
	✗ STAC	433	0.023	52.5

STAC	✓ COVER	609	0.15	23
	✗ COVER	259	0.11	8.1
	✓ COMPOSITE	727	0.15	20.5
	✗ COMPOSITE	141	0.07	7.21

**STAC input: filtered data regions found by**	**overlap?**	**no. of regions**	**mean(freq)**	**median(conf)**

COVER	✓ STAC	294	0.068	30.2
	✗ STAC	321	0.020	37.6

COMPOSITE	✓ STAC	297	0.14	23.1
	✗ STAC	313	0.045	65.2

STAC	✓ COVER	585	0.14	28.1
	✗ COVER	69	0.07	16.1
	✓ COMPOSITE	595	0.14	26.8
	✗ COMPOSITE	59	0.06	20.2

✓ - overlap

✗ - no overlap

This table shows a summary of the results obtained from comparing COVER/COMPOSITE to GISTIC.

We also investigated if the relative performance of STAC would improve if we manually filtered out individual regions with lower confidence scores. We decided to use only individual regions whose confidence scores were above the median confidence score of all reported regions. Using this filtered input, STAC identified 654 significant windows. Using *u *= 2 and *c *= 70th percentile for COVER and the 93.5th percentile for COMPOSITE, we identified a similar number of common regions (615 for COVER and 610 for COMPOSITE). Table 2b summarizes the results of this comparison and our conclusions are similar to those with the unfiltered input data.

We conclude that COVER and COMPOSITE are able to detect the majority of the regions found by STAC, and in addition they also detect common high-confidence CNV regions that occur in a smaller number of subjects that were missed by STAC.

## Implementation

The methods are implemented in an R package cnvpack. The main input is a list of detected individual CNV regions with the following information: Sample name, chromosome number, detected integer copy number, start and end genomic locations and a confidence score. The package can be downloaded from http://www.meb.ki.se/~yudpaw.

### Downstream analyses

#### CNV-association analysis

One important use of the identified common CNV regions is for group comparisons in association studies. For each region we test whether certain CNVs are over-represented in one group compared to the others. Typically, the Fisher's exact test or chi-squared test for contingency tables can be used. The test can be carried out for all identified common CNV regions and the issue of multiple testing can be dealt with using the false discovery rate (FDR) assessment. (See [Additional file 1] on how to use the package for such analyses.)

As an illustration we performed an association analysis on the common regions identified in the 112 control subjects using the optimal parameters for COVER and COMPOSITE. The subjects were grouped by ethnicity (YRI, CHBJPT and CEU). Both methods showed that there were a number of highly-significant CNV regions with p-value < 1e-06. Two of these regions were detected by both methods. The first one is a 16.2 kb deletion in chromosome 2 (genomic positions 203,004,035 to 203,020,242). This region occurs exclusively in the Yoruba population (17/37) and overlaps with the BMPR2 gene that has been linked to primary pulmonary hypertension [22]. The second region is a 4.6 kb deletion in chromosome 4 (genomic positions 20,982,707 to 20,987,259) that occurs among Yoruban (19/37) and CHBJPT (4/29). This region overlaps with the KCNIP4 gene that is known to interact with presenilin, a protein that has been reported to be involved in early-onset Alzheimer's disease [23].

#### Principal component analysis of CNV profiles

**Figure 7:**
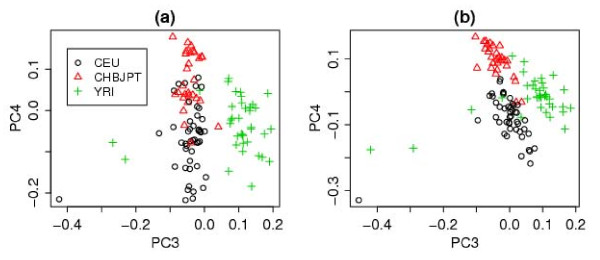
**The third and fourth principal components**. (a) Using COVER (with *u *= 3 and *c *= 60). (b) the same as (a) but using the output of complete-linkage CLUSTER (with cluster.limit = 0.6).

We also perform principal component analyses (PCA) to obtain informative plots of population differentiation in the CNV profiles (see [Additional file 1] for more information). For the HapMap samples, the first two components obtained using the optimal COVER parameters separate the Yoruba population (YRI) from the Caucasian(CEU) and Asian(CHBJPT) populations, but the other two populations are not very well separated (Figure S1 in the [Additional file 1]). A better separation between the CEU and CHBJPT populations is achieved using the third and fourth components(see Figure 7(a)) and the separation is further improved when we use CLUSTER to refine the CNV regions identified by COVER (Figure 7(b)).

## Conclusions

We have described and compared two different methods for identifying common CNV regions. Using 112 HapMap samples, we have shown that these methods produce common CNV regions that mostly follow Hardy-Weinberg equilibrium (HWE). For the eight HapMap samples where we compared the regions we identified to the reference CNV regions found by sequencing [10], the discordance rates can be as high as 80%, but this can be reduced to 60% by considering CNVs with higher confidence scores, thus showing the importance of further processing of the CNVs. The high level of discordance itself might be due to an inherent limitation in the SNP array as the platform for CNV detection, but perhaps also due to imperfection in the sequencing-based results. Further works are needed to explain the discordance level.

When we compared our methods to previously published methods, STAC and GISTIC, we found that our methods are better at identifying low-frequency CNVs. Moreover, STAC is rather rigid and insensitive to the actual breakpoints of a CNV region, because if two consecutive windows are reported as significant, we do not know if there is one large CNV which spans both windows, or two separate and distinct CNVs. Although we can decrease the window size to increase the resolution, in practice, decreasing the window size beyond a certain point will incur too much computational burden. Another limitation of previous methods is the lack of consideration of individual-specific confidence scores. This means that all samples contribute equally to the calculation of the statistic used to identify the common regions, while in fact, there is bound to be inter-sample variability, where some CNVs are more likely to be true positives than others.

The results of COVER and COMPOSITE are similar in terms of discordance rates and HWE violation rates, but COMPOSITE appears to be better at identifying rare regions. The HWE violation rates are useful in determining the choice of parameter values for COVER and COMPOSITE. For this particular data set, we observed a steeper reduction in HWE violation rates when we used COVER with a confidence score threshold set above the median or higher. For COMPOSITE, a more noticeable reduction in HWE violation rates was observed when we set *v *to the 94th percentile. For a new dataset, we encourage users to choose the confidence score and COMPOSITE score parameter thresholds for which steeper reduction in HWE violation rates can be observed.

When using COVER, the minimum number of individuals inside a common region (*u*) needs to be specified as well. If we are interested in rare variants in addition to the common variants, then it makes sense to set *u *= 1. Otherwise, *u *≥ 2 should be used. A higher *u *will result in the identification of fewer, but more highly-recurrent CNV regions. In our experience with the HapMap samples, clustering results produce better separation of the ethnic groups than indicated by the initial common CNV regions. In comparison with the highly-validated CNVs from Conrad et al. [17], the concordance rate of COVER also improves after refinement with CLUSTER. So, in summary, we recommend users to further refine the identified common CNV regions using CLUSTER.

## Authors' contributions

TSM and AS contributed equally to this work; TSM, AS, SC and YP conceived the study, performed data analysis and wrote the manuscript. KCS and CKS conceived the study. All authors read and approved the final manuscript.

## Supplementary Material

Additional file 1 The supplementary report documents details on how to use the R package cnvpack for the various analyses described in this paper. Click here for file

Additional file 2 This table shows details of the regions found by COVER. Click here for file

Additional file 3 This table shows details of the regions found by COMPOSITE. Click here for file
