# Supplementary material for: Identification of recurrent regions of copy-number variants across multiple individuals
Source: BMC Bioinformatics. 2010 Mar 22;11:147. doi: 10.1186/1471-2105-11-147 (PMC2851607; doi:10.1186/1471-2105-11-147)
Supplement: Additional file 1 — The supplementary report documents details on how to use the R package cnvpack for the various analyses described in this paper. [file 1471-2105-11-147-S1.DOC]

**Supplementary information for**

**Identification of common copy-number variation (CNV) regions using high-density SNP array**

**Teo Shu Mei, Agus Salim, Stefano Calza, Ku Chee Seng, Chia Kee Seng, and Yudi Pawitan**

The following R commands show how users can use the *cnvpack* package to identify common copy-number regions among 112 HapMap samples that are part of the Illumina iControl Database. The input files are PennCNV outputs based on Illumina 1M beadchip. We will only analyze common CNV regions (CNVR) detected in chromosome 1 to 22.

Download and install the *cnvpack* from <http://www.meb.ki.se/~yudpaw>

After installation, load the package in R using:

require(cnvpack)

The main functions in the package are summarized here:

1. read.cnv – reads in outputs from any CNV-detection software.
2. setreg – sets up common CNV regions using one of the three proposed methods, using user-specified parameters. The output will contain a list of common CNV regions and for each individual, a list of individual-specific common CNV regions and the discrete copy number calls.
3. plot – displays clusters of regions within each common region: only available when CLUSTER method is used.
4. hwe.cnv – tests Hardy-Weinberg Equilibrium (HWE) of diallelic CNV regions.
5. summary – prints descriptive statistics of size and frequencies of common CNV regions, conducts CNV-association analysis. The output is a list of common CNV regions with their frequency distribution and p-values from the association analysis, adjusted for multiple testing.

We read in the PennCNV output (input="penncnv") which contains the list of detected individual CNVs for the 112 HapMap samples. This file contains confidence score values for the individual CNVs (conf = TRUE), as well as the list of genes within the individual CNVs (annotated=TRUE). The read.cnv function will then return a data frame of class cnv.

out<-read.cnv(filename="112hapmap.txt",conf=TRUE,annotated=TRUE,input="penncnv",sep="\t")

Note that if the output is from PennCNV or QuantiSNP, there must be a column indicating confidence scores.

Outputs from other software can also be read in, provided it contains the following information in tab delimited columns: (1) chr: chromosome where CNV region is located, (2) sample: sample name, (3) cn: the detected integer copy-number with cn=2 indicating normal copy number, (4) startsnp: SNP or copy-number probe where the region starts and (5) endsnp: SNP or copy-number probe where the region ends and (6) confidence (optional): a score that reflects how confident the CNV-detection algorithm is in calling the integer copy number. The magnitude of the score reflects the level of confidence. If confidence scores are not available, set conf=FALSE.

Next, we load the Illumina IM beadchip annotation file and the phenotype data for our samples. The annotation file contains a list with components “Name”, “Position", "Chromosome" and "Chr.num"; “Name” contains the name of the SNP or CNV marker, “Position” contains the position of the markers in the genome , “Chromosome” specifies the Chromosome the marker is in (1,2,3,…,22, X, Y, XY, (M)itochondrial) and “Chr.num” is the numeric form of “Chromosome”, where “X” is coded as “23”, “Y” is coded as “24”, “XY” is coded as “25” and “M” is coded as “26”. The phenotype data is a data frame with columns “sample”, “gender” and “ethnic” (other covariates may be added).

load('ann_illumina1M.Rdata')

head(ann$Name)

[1] rs12354060 rs2691310 rs2531266 rs4477212 rs4124251 rs8179466

1072820 Levels: cnv10000p1 cnv10003p1 cnv10003p5 cnv10004p1 ... SNP98

head(ann$Position)

[1] 10004 46844 59415 72017 97215 224176

head(ann$Chromosome)

[1] 1 1 1 1 1 1

26 Levels: 1 10 11 12 13 14 15 16 17 18 19 2 20 21 22 3 4 5 6 7 8 9 M X ... Y

head(ann$Chr.num)

[1] 1 1 1 1 1 1

Next, we load the data frame containing gender and ethnicity information of the 112 samples.

load ('112hapmap_pheno.Rdata')

head(pheno)

sample gender ethnic

1 NA06985 FALSE CEU

2 NA06991 FALSE CEU

3 NA06994 TRUE CEU

4 NA07000 FALSE CEU

5 NA07029 TRUE CEU

6 NA07345 FALSE CEU

We now use the setreg function to set up the common CNV regions for these 112 individuals. The Cumulative Overlap Using Very Reliable Regions (COVER) method was used (method="COVER").The minimum acceptable confidence score is set to be equal to the 60^th^ percentile of all reported confidence scores(high.conf=60). Inside a genomic location in a common CNV region, there must be at least 3 individuals whose individual CNVs overlap with that genomic location (LIM=2), with the deletion and duplication regions considered simultaneously (cnv.abnormality= "both"). If confidence scores are not available, set high.conf = NA.

cnvr<-setreg(out,anno.list = ann,pheno.data= pheno,high.conf = 60,LIM = 2,method = "COVER", cnv.abnormality = "both")

The cnvr$reg$loc object containing the list of all identified common CNV regions with their positions in term of probes number:

head(cnvr$reg$loc)

$`limit=2`

st en st.chr regmin

1 5 47 1 -15

2 205 248 1 -7

3 269 283 1 -4

4 2681 2695 1 -4

5 4201 4208 1 -3

6 4367 4381 1 -5

7 5380 5392 1 -4

8 5395 5399 1 -3

.

.

.

443 1029556 1029574 22 -8

The cnvr$reg$regID is a list. For each individual, it contains the CNVR ID (corresponding the ID in cnvr$reg$loc) for which that individual has copy-number variation. The corresponding integer copy-number call is given in cnvr$reg$call. The ordering of the individual in cnvr$reg$regID corresponds to the way they are ordered in cnvr$pheno, so that cnvr$reg$regID[[1]] contains the CNVR for individual on the first row of cnvr$pheno; cnvr$reg$regID[[2]] contains the CNVR for individual on the second row of cnvr$pheno and so on.

**Test for Hardy-Weinberg Equilibrium**

We can test for Hardy-Weinberg Equilibrium of common diallelic CNV regions when cnv.abnormality= "both" in setreg.

cnvr.hwe = hwe.cnv(cnvr)

The main component of interest in cnvr.hwe would be cnvr.hwe$group1$fdr which gives the pvalue from testing HWE on each CNVR in cnvr$reg$loc, corrected for multiple testing using Benjamini-Hochberg method (the pvalue is set to NA for multiallelic CNVR).

head(cnvr.hwe$group1$fdr)

[1] 0.2992617 0.6346215 0.7874008 0.8400692 0.8400692 0.7874008

The ordering of the adjusted pvalues in cnvr.hwe$group1$fdr corresponds to the CNVR in cnvr.hwe$group1$reg, which in turns corresponds to cnvr$reg$loc.

head(cnvr.hwe$group1$reg)

[1] 1 2 3 4 5 6

**Downstream Analyses**

**Comparing between-group CNV frequency**

We can obtain a summary of the number of CNVs and distribution of CNV length for each level of group variable. Using test= TRUE, we can compare between-group CNV frequency. A output of the list of common CNVs with their profiles and frequency distribution as well as p-values and false discovery rates will be saved in LIST of CNV regions.xls (see Supplementary Table I) .Here, we are comparing between ethnicity, where the ethnicity information was located in the third column of our phenotype data (group= 3).

summary(cnvr,anno.list= ann,test= TRUE,group= 3)

A group-by-group summary of the CNV regions will be displayed as follows:

summary(cnvr,anno.list= ann,test= TRUE,group= 3)

CNV Region group-by-group summary:

group=CEU

Number of unique CNV regions across individuals = 322

Average length of CNV regions 71039.51

Median length of CNV regions 19175

Distribution of length

(0,1e+03] (1e+03,1e+04] (1e+04,5e+04] (5e+04,1e+06]

6 90 148 73

CNV Region group-by-group summary:

group=CHJP

Number of unique CNV regions across individuals = 175

Average length of CNV regions 106601.0

Median length of CNV regions 23551

Distribution of length

(0,1e+03] (1e+03,1e+04] (1e+04,5e+04] (5e+04,1e+06]

5 56 54 55

CNV Region group-by-group summary:

group=YRI

Number of unique CNV regions across individuals = 362

Average length of CNV regions 63749.46

Median length of CNV regions 18185

Distribution of length

(0,1e+03] (1e+03,1e+04] (1e+04,5e+04] (5e+04,1e+06]

5 102 176 74

Correlation of CNV Regions Frequency

group=CEU group=CHJP group=YRI

group=CEU 1.0000000 0.5093919 0.1071928

group=CHJP 0.5093919 1.0000000 0.2491267

group=YRI 0.1071928 0.2491267 1.0000000

**Principal Component Analysis (PCA) of CNV profiles**

To perform PCA on the CNV profiles, we need to create$A$, a $n\times p$ matrix of absence-presence, where $n$ is the number of individuals and $p$ is the number of CNV regions. The *j^th^* element of the *i^th^* row will be equal to 1 if the *i^th^* subject has CNV in the *j^th^* region and 0 otherwise. We can extract this information from the regID component of reg list, an output of setreg.princomp(t(x))will perform the analysis on $\frac{1}{p}AA^{T}$.A matrix of scatterplots of the first five principal component loadings is shown in Figure S1. For a clearer picture, we also plot loading 3 versus loading 2 in Figure S2. We can see that the three ethnic groups are well separated. A summary of the proportion of data explained by the different components can be obtained by using summary ( ). There are in total 112 principal component loadings, and the first 5 components explain about 27% of the variance in the data.

# The following commands perform PCA on integer copy-number

n = 112 #number of individuals

ncol = max(unlist(cnvr$reg$regID)) #get the largest CNVR ID

reg = unique(unlist(cnvr$reg$regID)) #get a vector of unique CNV region ID

x = matrix(2,nrow=n,ncol=ncol)

for(i in 1:n){ #for each individual

 x[i,unlist(cnvr$reg$regID[[i]])] = unlist(cnvr$reg$call[[i]])}

out = princomp(t(x))

#Figure S1

pairs(out$load[,1:5],main="First five principal component loadings",

col=as.numeric(factor(cnvr$pheno$ethnic)),pch=substring(cnvr$pheno[,3],3,3),cex = 0.6)

summary(out)

Importance of components:

Comp.1 Comp.2 Comp.3 Comp.4 Comp.5

Standard deviation 0.8847292 0.66110492 0.58680472 0.44996690 0.40621197

Proportion of Variance 0.1160093 0.06477583 0.05103398 0.03000775 0.02445556

Cumulative Proportion 0.1160093 0.18078518 0.23181916 0.26182690 0.28628247

Comp.6 Comp.7 Comp.8 Comp.9 Comp.10

Standard deviation 0.39876638 0.39198306 0.37636555 0.37189963 0.36877064

Proportion of Variance 0.02356727 0.02277230 0.02099384 0.02049858 0.02015510

Cumulative Proportion 0.30984974 0.33262203 0.35361588 0.37411446 0.39426955

.

.

.

Comp.11 Comp.12 Comp.13 Comp.14 Comp.15

Comp.108 Comp.109 Comp.110 Comp.111

Standard deviation 0.0758989870 0.0740548526 0.0701807397 0.0681210169

Proportion of Variance 0.0008537765 0.0008127917 0.0007299752 0.0006877561

Cumulative Proportion 0.9972214494 0.9980342411 0.9987642163 0.9994519725

Comp.112

Standard deviation 0.0608086094

Proportion of Variance 0.0005480275

Cumulative Proportion 1.0000000000

Figure S1. The first five principal component loadings based on the CNV profiles of the European population CEU (black), the Yoruba population (green) and the Asian population (red) based on CNV region profiles identified using COVER with u=3 and c=60.


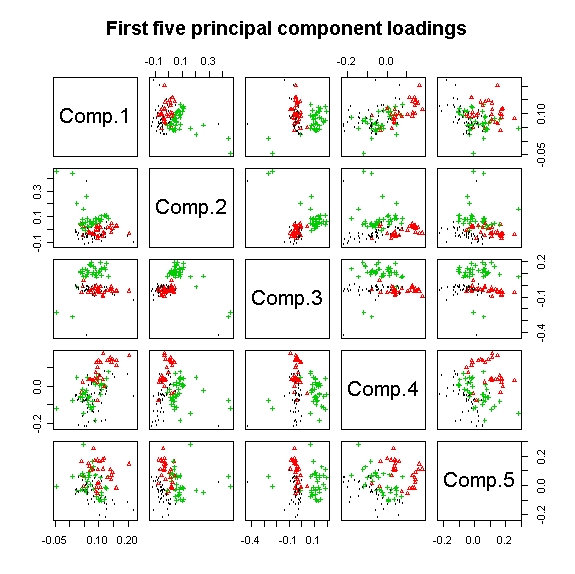


**COVER with CLUSTER**

We refine the CNV regions identified by COVER above and look for clustering of individual regions (see Figure S3 for evidence of clustering). Here, we use hierarchical clustering with complete linkage and require that within-cluster similarity to be at least 60% (see Table S1 for effects on linkages and cut-off on number of clusters).

cnvr <-setreg(out,anno.list= ann,pheno.data= pheno,high.conf= 60,LIM= 2,method= "COVER", cnv.abnormality= "both",cluster.method=’complete’,cluster.limit=0.6)

We perform principal component analysis (PCA) based on the common CNV region profiles identified by this configuration of threshold parameters.

## PCA

p = dim(cnvr$reg$loc[[1]])[1]

n = length(cnvr$reg$regID) #no. of individuals

x = matrix (2, nrow = n, ncol = p)

for(i in 1:n){ #for each individual

index = match(as.numeric(unlist(cnvr$reg$regID[[i]])),as.numeric(rownames(cnvr$reg$loc[[1]])))

x[i,sort(index) ] = unlist(cnvr$reg$call[[i]])

}

out = princomp(t(x))

## Figure S2

pairs(out$load[,1:5],main="First five principal component loadings",

col=eth,pch=eth,cex = 0.6)

Figure S2. The first five principal component loadings based on the CNV profiles of the European population CEU (black), the Yoruba population (green) and the Asian population (red) based on CNV region profiles identified using COVER with u=3 and c=60 and refined using CLUST with complete linkage and cluster.limit=0.6


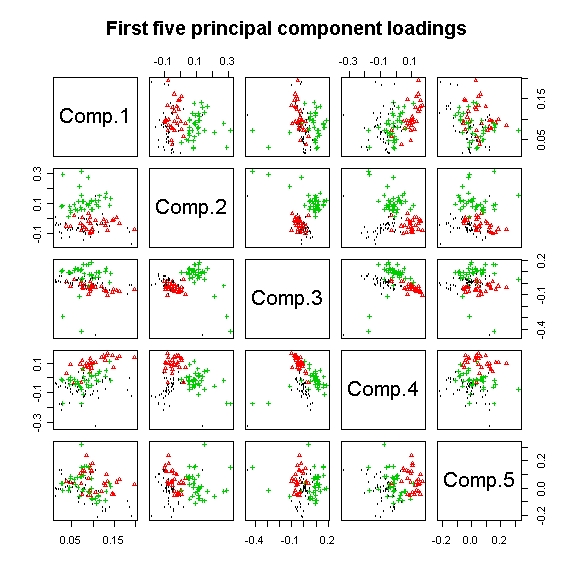


Figure S3. (a) A common CNV region identified using COVER with u=3 and c=60, exhibiting clusters of individual regions and (b) identified clusters of individual regions obtained using average linkage and 80% similarity cut-off.


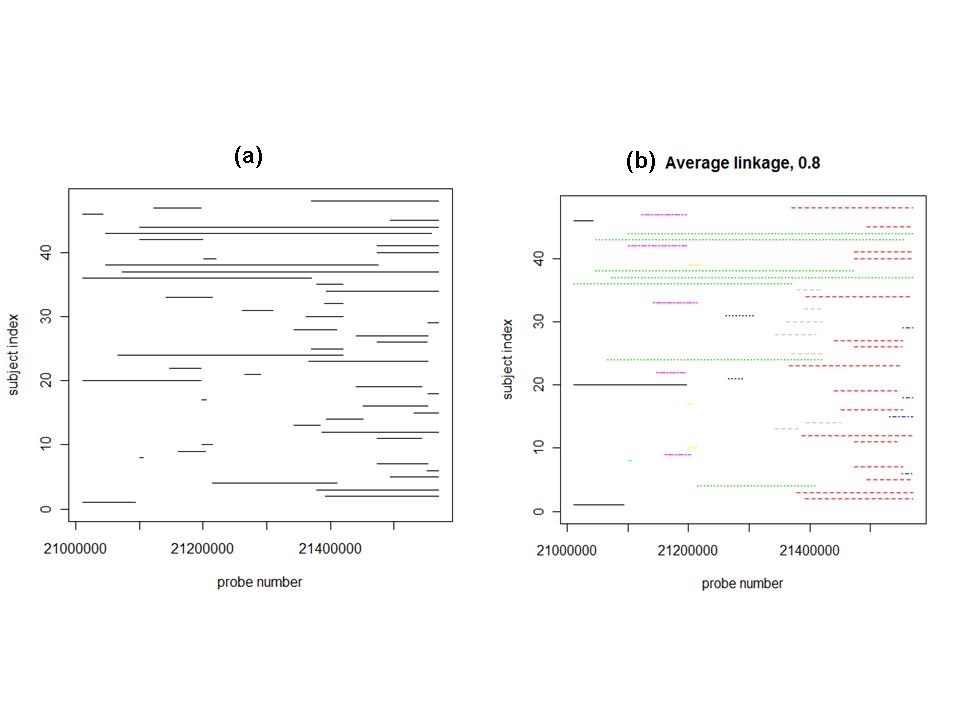


Figure S4: Illustration of COVER and COMPOSITE statistics: Thick lines indicate individual regions whose confidence scores are above a certain threshold *c*, (b) Illustration of COVER statistic, (c) Illustration of COMPOSITE statistic.


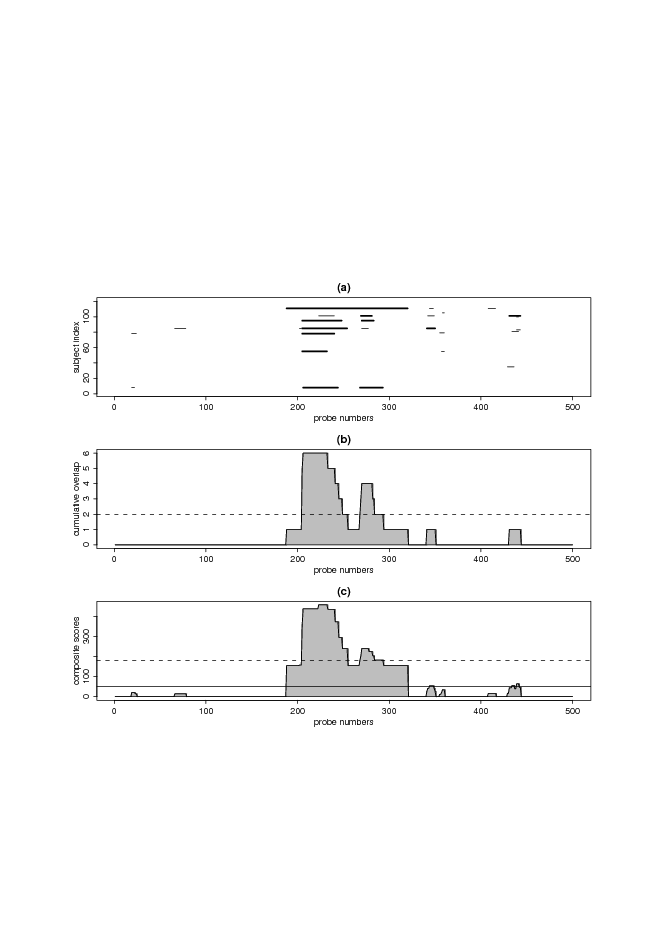


Table S1. The number of clusters identified in CNV region in Figure S3 as a function of dissimilarity (1-similarity) cut-off point and linkage.

| Dissimilarity cut-off | Number of Clusters | | |
| --- | --- | --- | --- |
|  | Single Linkage | Average Linkage | Complete Linkage |
| 0.01 | 1 | 2 | 9 |
| 0.05 | 2 | 5 | 9 |
| 0.10 | 2 | 6 | 10 |
| 0.20 | 2 | 9 | 12 |
| 0.30 | 4 | 12 | 14 |
| 0.50 | 11 | 17 | 21 |
| 0.60 | 16 | 21 | 23 |
| 0.70 | 24 | 27 | 28 |
